# Vat Polymerization by Three-Dimensional Printing and Curing of Antibacterial Zinc Oxide Nanoparticles Embedded in Poly(ethylene glycol) Diacrylate for Biomedical Applications

**DOI:** 10.3390/polym15173586

**Published:** 2023-08-29

**Authors:** Guy Naim, Netta Bruchiel-Spanier, Shelly Betsis, Noam Eliaz, Daniel Mandler

**Affiliations:** 1 Institute of Chemistry, The Hebrew University of Jerusalem, Jerusalem 9190401, Israel; guy.naim3@mail.huji.ac.il (G.N.); netta.spanier@mail.huji.ac.il (N.B.-S.); shelly.betsis@mail.huji.ac.il (S.B.); 2 Department of Materials Science and Engineering, Tel-Aviv University, Tel Aviv 6997801, Israel; neliaz@tauex.tau.ac.il

**Keywords:** zinc oxide nanoparticles, 3D printing, digital light processing (DLP), antibacterial properties, UV curing, implants

## Abstract

Digital light processing (DLP) is a vat photopolymerization 3D printing technique with increasingly broad application prospects, particularly in personalized medicine, such as the creation of medical devices. Different resins and printing parameters affect the functionality of these devices. One of the many problems that biomedical implants encounter is inflammation and bacteria growth. For this reason, many studies turn to the addition of antibacterial agents to either the bulk material or as a coating. Zinc oxide nanoparticles (ZnO NPs) have shown desirable properties, including antibacterial activity with negligible toxicity to the human body, allowing their use in a wide range of applications. In this project, we developed a resin of poly(ethylene glycol) diacrylate (PEGDA), a cross-linker known for its excellent mechanical properties and high biocompatibility in a 4:1 weight ratio of monomers to water. The material’s mechanical properties (Young’s modulus, maximum elongation, and ultimate tensile strength) were found similar to those of human cartilage. Furthermore, the ZnO NPs embedding matrix showed strong antibacterial activity against *Escherichia coli* (*E. coli*) and *Staphylococcus aureus* (*S.A.*). As the ZnO NPs ratio was changed, only a minor effect on the mechanical properties of the material was observed, whereas strong antibacterial properties against both bacteria were achieved in the case of 1.5 wt.% NPs.

## 1. Introduction

Three-dimensional printing is an additive manufacturing method that is often used for medical applications to fabricate complex structures, such as surgical implants, with high customizability [1]. Digital light processing (DLP) is a common 3D printing technique that is often used for medical applications. DLP enables the incorporation of a variety of materials into the printed resin, thus tuning the implant properties [2]. Medical implants are required to possess certain biophysical and biochemical properties mimicking the replaced natural organs, such as elasticity, strength, and durability. 

Orthopedic transplantations are especially susceptible to infections, which might develop during the surgical process or due to contaminations originating from the surrounding tissues. In both cases, this can lead to biofilm formation, and thus—to implementation failure—resulting in additional expensive procedures [3]. Therefore, ideal orthopedic implants should also exhibit antibacterial activity to overcome infections by common pathogens such as staphylococci [4].

Antibacterial activity comprises the eradication of bacteria or the suppression of their ability to reproduce [5]. Hence, medical implants possessing localized antibacterial activity should replace the wide usage of antibiotics administrated to patients undergoing orthopedic operations. Such antibacterial activity can be obtained in two distinct ways—either by coating the surface of the implant or by embedding the antibacterial substance within the implant. The first approach, in which either antibiotics or antibacterial nanoparticles (NPs) are accommodated in the coating, is currently more common. Excellent reviews have described this approach [6,7,8,9].

The alternative approach, where the antibacterial substances are incorporated into the implant, is more challenging; however, offers advantages such as high durability and resistance to abrasion. Three-dimensional printing, and specifically DLP, seems to be an ideal approach for the formation of medical implants with embedded antibacterial agents. A literature survey reveals that the usage of localized antibiotic agents or drug delivery in 3D-printed implants is well documented [2,3,10]. Yet, there are continuous attempts to replace antibiotics and their adverse effect with a zero-emission approach in which non-leaching antibacterial substances are part of the implant. An appealing alternative is the family of inorganic NPs. The addition of NPs into printable resins has been described [1,3,11,12]; however, only a few papers dealing with the introduction of NPs into the printed implants’ resin have been published [13,14,15,16]. For example, Bsat et al. incorporated hydroxyapatite and titania NPs into poly(methylmethacrylate) printed implants that showed efficiency as load-bearing implants. Cao et al. showed that zirconia/hydroxyapatite composite scaffold has potential implications for bone repair. Zou et al. used poly(lactide-co-glycolide) with zinc-based zeolitic imidazolate frameworks to show reduced inflammatory cell infiltration in infected rat implants. Chang et al. reported the usage of inorganic radiopaque nanofillers, including barium sulfate, bismuth subcarbonate, and bismuth oxychloride in DLP resin, for monitoring the degradation of medical implants.

One of the most promising inorganic NPs possessing high antibacterial activity while being harmless to humans is zinc oxide (ZnO). The mechanism of the antibacterial activity of ZnO NPs is not clear yet [17]. Three mechanisms have been suggested: generation of reactive oxygen species (ROS), leaching of zinc ions, and direct contact with the bacteria’s cell membrane. Unlike traditional antibiotic substances, the generality of such NP antibacterial mechanisms toward biomolecules suggests the reduction or elimination of more resistant bacteria [18]. Moreover, the versatility of NP synthesis to generate different shapes and sizes contributes greatly to the increased interest in such NPs due to the non-specific activity of such inorganic antimicrobial agents [19]. 

Despite the uncertainty in the ZnO antibacterial mechanism, studies suggest that relatively low concentrations of these NPs have a significant antibacterial effect and thus can be used successfully for medical applications. Even though ZnO is classified as non-hazardous by the US Food and Drug Administration (FDA), a zero-emission approach where leaching of the ZnO NPs or Zn(II) ions is avoided while maintaining high antibacterial activity is preferred.

Poly(ethylene glycol) diacrylate (PEGDA) and PEGDA-based hydrogels are highly tunable cross-linked materials that act as biocompatible substrates and can carry certain materials in vivo. Furthermore, as cross linkers, they offer certain rigidity and mechanical properties that can be tailored to the physiologically relevant range of cells in 3D printing, showing good biocompatibility in a wide range of biomedical applications [20,21,22]. The mechanical properties of the hydrogels and their behavior when swelling, as well as their biochemical properties, can be controlled by varying the molecular weight or concentration of the polymer or by the addition of metal oxide NPs that stabilize the structure [23,24]. For all those reasons, the addition of ZnO NPs to a PEGDA resin shows great promise, as the resin can be optimized in numerous ways, including the addition of different copolymers, controlling the molecular weight of the components, changing the shape and sizes of the NPs or by studying the effects of different printing parameters on the resulting substrate. 

The usefulness of PEGDA for osteochondral tissue (including cartilage) regeneration and treatment is well-studied and reviewed, as it allows cell encapsulation in a highly hydrated environment equivalent to the native microenvironment [25,26]. For example, Wang et al. successfully created a bioprintable PEGDA-based resin that promoted cellular activities in cartilage-specific microenvironments, as confirmed by cell viability and biochemical studies [27]. Hao and coworkers designed a self-assembled DLP resin with micro-patterned scaffolds of PEGDA biomaterial and demonstrated how cells engraft into the porous PEGDA, presenting a possible cartilage reconstruction strategy [28]. 

**Scheme 1:**
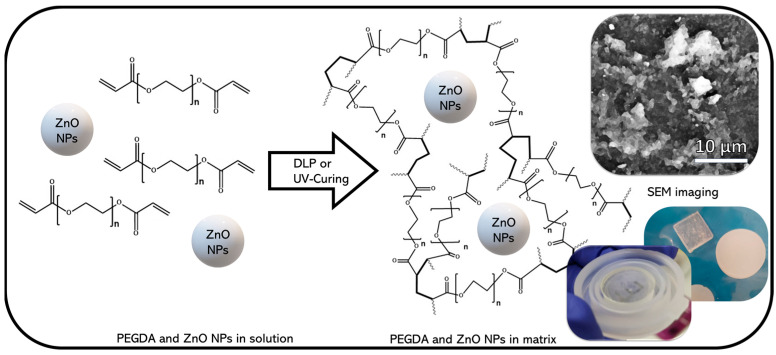
Schematics of the printed and cured vat photopolymerization of antibacterial PEGDA embedding ZnO NPs. The bonds that are formed upon polymerization are shown in bold.

Here, we report two types of vat polymerization of ZnO NPs embedded in PEGDA, as seen in Scheme 1. Specifically, DLP printed and UV-cured PEGDA matrices with different levels of ZnO NPs were compared in their mechanical and antibacterial performance. We found that DLP 3D printed with 1.5–2 wt.% ZnO NPs resulted in appropriate properties for cartilage implants, i.e., Young’s modulus, *E*, of 11.7 ± 0.6 MPa, maximum elongation of 10.9–14.6%, and ultimate tensile strength of 1.3 ± 0.3 MPa. Furthermore, the implants exhibited a very high antibacterial activity. 

## 2. Experimental Section

### 2.1. Materials and Equipment

Most chemicals were purchased from Sigma-Aldrich (St. Louis, MI, USA): poly(ethylene glycol) diacrylate (PEGDA, average Mn = 700), ZnO NPs (≤40 nm average particle size, 20 wt.% in H_2_O), sodium phosphate monobasic (AR), sodium phosphate dibasic (AR), sodium chloride (AR), Zincon monosodium salt. The following chemicals and kit were ordered from Thermofisher Scientific (Bleiswijk, The Netherlands): BACTO agar, BACTO yeast extract, and BACTO tryptone. Live\Dead measurements were carried out using a LIVE/DEAD BacLight bacterial viability kit for microscopic analysis. The photoinitiator, diphenyl(2,4,6-trimethylbenzoyl)phosphine oxide (TPO), was purchased from TCI Europe N.V. The bacteria *Escherichia coli* (*E. coli*) and *Staphylococcus aureus* (*S.A*.) were cultivated in-house. The UV light lamps (M405L4-C1—405 nm, 365 nm) were purchased from Thorlabs (Bergkirchen, Germany). An Asiga Pico 2 3D printer (Alexandria, Australia) was used for all printing. An orbital shaker incubator (MRC, Holon, Israel) was used for cultivating the bacteria, and a 3150 Tuttnaur autoclave was used for sterilization. The mechanical properties were measured with an Instron 3345 (Instron, Norwood, MA, USA). A high-performance scanning electron microscope (SEM) equipped with an energy-dispersive X-ray spectroscopy (EDS) detector (Apreo 2 and UltraDry, respectively, Thermo Scientific, Waltham, MA, USA) was used for imaging the surfaces, while an FV-1200 confocal microscope (Olympus, Tokyo, Japan) was used for live–dead measurements.

### 2.2. Formula Preparation

In a typical experiment, separate PEGDA and ZnO NP solutions in three distilled water (TDW) were prepared. Then, an exact mass of the diluted solution of ZnO NPs was dropwise added to the PEGDA solution while stirring to obtain a final 80 wt.% of the PEGDA. TPO was added as the photoinitiator (0.1 wt.% of dry TPO for UV curing or 0.015 wt.% for DLP printing) to the PEGDA/ZnO NPs solution. The solution was covered and mixed for 20 min at 950 rpm and 30 ± 3 °C. The 4 most repeated concentrations are summarized in Table S1.

### 2.3. UV Curing and DLP Printing

For UV curing, the solutions were vortex-stirred for 1 min, followed by pouring into a mold with either a doggybone shape (1.7 mL) or a 13 mm diameter disk shape (0.4 mL). The molds were exposed to 405 nm UV light (30 ± 3 mW) for 20 min. For DLP printing, 30 mL solution was placed inside the vat, and the specific printing parameters were adjusted for each solution within the ranges in Table S2. Once the polymerization process was completed, the samples were washed with TDW and dried with an absorbent paper. The thickness of both the UV-cured and the 3D-printed samples was kept at 3 mm. The molds used for UV curing were replaced every 6 months as continuous usage damaged the molds over time, a problem 3D printing does not face as every sample is made directly on the printing plate. 

### 2.4. Antibacterial Tests

A standard phosphate-buffered saline (PBS) solution, pH 7.4, was used for the antibacterial tests. The solution was filtered using a 0.22 μm syringe filter and sterilized in an autoclave. A lysogeny broth (LB) solution was also sterilized in the autoclave.

The antibacterial activity was tested as described by us elsewhere [29], with some small modifications. Specifically, colonies of *E. coli* and *S.A*. were cultivated at 37 °C while shaking 15 mL of LB at 120 rpm overnight. The dead bacteria were removed from the suspension using centrifugation (4000 rpm for 10 min) and washed with PBS three times. The remaining living bacteria were dissolved in 10 mL PBS using a vortex. The cell density of the bacterial suspension was adjusted to 106 cfu/mL by adding PBS to the cultivated suspension. 

Before the addition of bacteria at the beginning of each test, the plate and the disk-shaped samples were exposed for 3 min to a high flux of 365 nm UV light for sterilization. A 5 μL aliquot of bacterial suspension was placed on top of each tested sample and allowed to incubate for 4 h in a twelve-well plate while agitating at 120 rpm and 37 °C. This was essential to ensure that all the bacteria in the suspension were exposed to the UV-cured and 3D-printed polymeric surfaces. During this process, the major antibacterial activity tested takes place. After 4 h, the substrates were submerged by adding 2.5 mL of LB and incubated under the same conditions (120 rpm, 37 °C) for an additional 18 h. During this process, the surviving bacteria can proliferate, increasing their concentration to a measurable level. Lastly, a known volume of the suspension was diluted in fresh LB and analyzed spectrophotometrically (at 600 nm, using a fresh LB as a reference) to verify bacterial proliferation. The viability percentage was estimated as previously reported [30]:

(1) $$Viability\;\%=\frac{O.D._{s}}{O.D._{c}}\cdot 100\%$$

where *O.D._s_* and *O.D._c_* are the average optical densities of the sample and the control, respectively. The control experiments were performed identically; however, they did not involve any ZnO NPs. Each assay was performed four times. The control group was tested for every individual twelve-well plate, as the minor differences (such as the location placed in the incubation or time under UV light for sterilization) between them affected the result. Live/Dead procedure was carried out according to the manufacturer’s instructions; however, no statistical analysis was carried out using those results due to the lack of a decent number of detected bacteria post-process (Figure 4).

### 2.5. Mechanical Property Measurements

The Young’s modulus (*E*), ultimate tensile strength, and maximum elongation at the breakpoint of the samples were measured using doggybone samples (Figure S1). The same shape was made by DLP printing and UV curing. The samples were tested at a tension speed of 5 mm/min and at room temperature.

### 2.6. Mesh Size

The mesh size (ξ) is defined as the maximum size of solute that can freely pass through the matrix [31]. Accordingly, particles with larger dimensions can either be trapped in the polymer or slowly diffuse in and out at a slow pace. Calculating the mesh size can be accomplished using the swelling index (*Q*), the Flory–Huggins polymer–solvent interaction parameter (χ, assumed to be equal to 0.426, as used in previous studies on PEGDA), the polymer volume fraction in the relaxed and swollen states ($v_{2,r},v_{2,s}$), the weight ratios, and the appropriate material densities [32,33].

*Q* was calculated using Equation (2):

(2) $$Q=\frac{W_{s}-W_{d}}{W_{d}}$$

where $W_{d}$ and $W_{s}$ are the weights of the same dried and soaked sample, respectively. Specifically, the samples were dried for 4 h at 120 °C, followed by soaking in PBS for 24 h at room temperature (no significant change in weight was observed after another week in PBS) and dried using Kimwipes. 

$v_{2,r}$ was calculated in the precursor solution using Equation (3):

(3) $$v_{2,r}=\frac{V_{PEGDA}}{V_{sol}}=\frac{m_{PEGDA}}{m_{sol.}}\cdot \frac{\rho_{sol.}}{\rho_{PEGDA}}$$

$v_{2,s}$ was obtained using Equation (4):

(4) $$v_{2,s}=\frac{1}{Q\cdot \frac{\rho_{PEGDA}}{\rho_{TDW}}+1}$$

where $m_{PEGDA}$ and $\rho_{PEGDA}$ and $m_{sol.}$ and $\rho_{sol.}$ are the masses and densities of the polymer (PEGDA) and the solution, respectively, and $\rho_{TDW}$ is the density of TDW.

The average molecular weight of two consecutive crosslinks ($M_{c}$) was found using Merrill’s modification to Florry’s derivation [34], as expressed by Equation (5):

(5) $$\frac{1}{M_{c}}=\frac{2}{M_{n}}-\frac{\ln\left(1-v_{2,s}\right)+v_{2,s}+\chi \cdot v_{2,s}{}^{2}}{V_{1}\cdot \rho_{sol.}\cdot v_{2,r}\cdot \left[{\left(\frac{v_{2,s}}{v_{2,r}}\right)}^{\frac{1}{3}}-\left(\frac{v_{2,s}}{2\cdot v_{2,r}}\right)\right]}$$

where $M_{n}$ is the average molecular weight of the starting polymer (700 g/mol), and $V_{1}$ is the molar volume of water (18 cm^3^/mol). Lastly, ξ can be calculated following Equation (6):

(6) $$\xi =l\cdot \sqrt{2\frac{M_{c}\cdot C_{n}}{M_{r}}}\cdot v_{2,s}{}^{-1/3}$$

In this case, l is the weighted average length of one carbon–carbon bond and two carbon–oxygen bonds, $M_{r}$ is the molecular weight of the PEG repeating unit (44 g/mol), and $C_{n}$ is the characteristic ratio for the PEG unit. l and $C_{n}$ equal 1.5 Å and 4, respectively [35].

### 2.7. Leaching out of Zinc Ions

The leaching of zinc ions was measured using a published spectroscopic method [36]. Specifically, a Zn(II) complexing agent, i.e., Zincon, was dissolved (1.6 mM) in water and kept in the refrigerator until use. Printed and UV-cured disk-shaped samples with different concentrations of ZnO NPs were placed in 5 mL PBS. The concentration of Zn(II) was measured every few days for 3 months according to the following procedure. For each measurement, a sample of 850 μL was mixed with 100 μL of borate buffer (0.5 M at pH 9.0), stabilizing Zn(II) in their ionic form, and 50 μL of the Zincon solution. The absorbance at 615 nm was recorded and compared to a calibration curve, which was constructed according to a similar procedure with known concentrations of ZnCl_2_.

### 2.8. Statistical Analysis

Data are presented as mean ± standard error of the mean. At least three samples and up to seven samples were used to determine the presented averages.

## 3. Results and Discussion

Samples made of ZnO NPs embedded in PEGDA were formed by both 3D printing and UV curing. The NPs were added as a means of imparting antibacterial activity and improving the mechanical properties. We aimed to compare the samples prepared by the two methods and optimize the concentration of the embedded ZnO NPs.

### 3.1. ZnO NP Distribution in Samples

**Figure 1:**
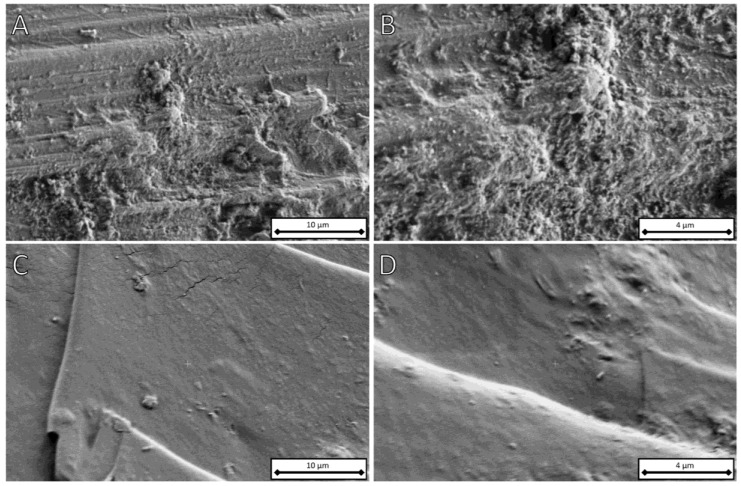
SEM images of samples containing 1.5 wt.% ZnO NPs made by (**A**,**B**) DLP printing and (**C**,**D**) UV curing.

Figure 1 and Figure S2 are SEM and optical microscopy images, respectively, of samples prepared by 3D printing and UV curing. Whereas the SEM images do not show any significant differences between the 3D printed and the photopolymerized samples, optical microscopy shows differences in the hundreds of microns scale, which is the result of the method of preparation. Hence, for example, the top surface of the DLP printed sample was touching the printer surface, and therefore, all samples have similar scratches. Yet, the cross-sections of both printed and UV-cured samples are the same. Table 1 and Figure S3 summarize the results of the EDS analysis of samples containing 1.5 wt.% ZnO NPs. The polymer seems homogeneous throughout the two samples, implying that the polymerization reaction happened approximately at the same pace in all depths of the mold and layers of the print. It is plausible that larger molds (used for UV-curing) will produce a less homogeneous structure as the absorbance and scattering of light in the top layers might affect the photoinitiation process in deeper parts of the mold. Using a DLP printer, on the other hand, is not likely to cause such problems because each layer is directly exposed to UV light during the printing process.

Figure S3 shows the EDS mapping of samples containing 1.5 wt.% ZnO. The results are summarized in Table 1. It is evident that O and C are the most commonly exposed atoms on the surface, as expected for PEGDA. The Zn content in both samples varies between 2.0 and 2.8 wt.% after the removal of water by vacuum. This closely matches the calculation that yields 2.3 wt.%, based on the initial concentration of ZnO (without water) in the polymerization solutions. While some spots with high concentrations of Zn are found containing up to 40 wt.%, scanning over the surface of the sample reveals a Zn distribution between 1.7 and 5.6 wt.%. Thus, it can be concluded that the NPs are quite homogeneously distributed across the samples with no noticeable aggregation. Moreover, the distribution of the ZnO NPs leaves no space for a colony of bacteria to thrive on top of the implant without close contact with the NPs. 

### 3.2. Leaching of Zn(II) Ions and Mesh Size

It is of utmost importance to ensure that the NPs embedded in a medical implant do not leach out, which might affect the nearby organs. Therefore, we studied the stability of the ZnO NPs by measuring the levels of Zn(II) leaching out of the samples, both as ions and as NPs, as a function of the immersion time in PBS. The analysis was carried out using Zincon, which is known to selectively bind Zn^2+^ ions. The detection limit of this method is ca. 2.5 μM of Zn(II). No noticeable leaching was observed for any sample immersed in the PBS for up to 3 months. Since the samples contained up to 22 mg of ZnO NPs (for 2 wt.% ZnO NP samples) and were placed in 5 mL of PBS, it implies that less than 0.004% of zinc leached out. Therefore, we can infer that the ZnO NPs are strongly bound within the printed and UV-cured samples, suggesting that such matrices could be used in vivo. 

**Figure 2:**
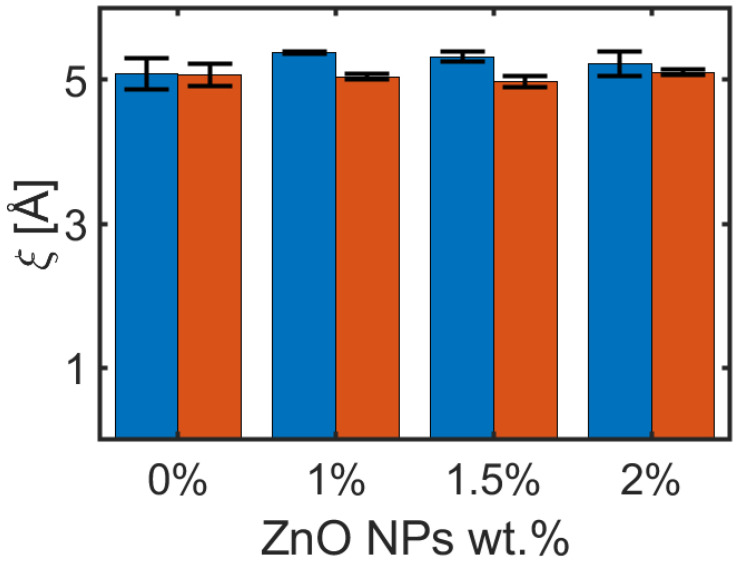
Calculated mesh size, ξ, for UV-cured (blue) and DLP-printed (red) samples for different concentrations of ZnO NPs.

Figure 2 depicts the calculated mesh size, ξ, as a function of the concentration of ZnO NPs in the matrix. We recall that ξ is a measure of the maximum size of solute that can freely pass through the matrix. Further description of ξ and the relevant formulas can be found in the Supporting Information. It is evident that ξ is almost independent of either the concentration of the ZnO NPs or the vat photopolymerization method. In all tested cases, the leakage of ZnO NPs out of the polymer is unlikely because of their diameter (ca. 40 nm), which is 80 times larger than ξ (ca. 5 Å). On the other hand, Zn(II) ions are expected to diffuse across the matrix considering their smaller diameter (ca. 1.8 Å for a coordination number of 8 [37], or 8.6 Å including the hydrated diameter [38]). Therefore, it seems that the leaching of Zn^2+^ occurs to only a negligible extent while ZnO NPs are entrapped in and on top of the sample.

Hence, we expect that the entrapment of ZnO NPs inside the matrix will result in a continuous antibacterial effect. We recall that the mechanism of antibacterial activity of ZnO involves either the membrane destruction of the bacteria, the formation of short-living ROS, or Zn^2+^ poisoning. The first two mechanisms require direct or very close contact between the ZnO and the bacteria, and therefore, we expect to detect antibacterial activity as long as exposed ZnO NPs are present on the sample’s surface. Moreover, the strong binding of the ZnO NPs to the matrix could negate expected tissue toxicity, as the NPs cannot freely travel out of the sample, and Zn^2+^ poisoning depends on the leaching of the ions from the ZnO NPs. Such leaching is not anticipated, given the aforementioned results.

### 3.3. Mechanical Properties

The introduction of ZnO NPs might affect the mechanical properties of the samples by stabilizing the hydrogel structure or by causing certain defects to the composite. Therefore, we measured Young’s modulus, *E*, the maximum elongation, and the ultimate tensile strength of both 3D-printed and UV-cured samples (Table 2). *E* was obtained by linear fitting of the elastic portion of the stress–strain plots, whereas the maximum elongation and ultimate tensile strength were derived from the plastic region of the same graph.

The Young’s modulus found for the samples matches the physiological range of the human cartilage, which changes over age and between different people, ranging from 3.6 to 12 MPa, with noticeable degradation with age [39]. Finetuning the specific mechanical properties for each individual can then be customized through several different techniques, including tuning the printing parameters to include gradient porosity or density or using selectively sieved salt particulates as composite in inks [40,41,42,43].

Therefore, customized DLP-printed implants created from such resins could be used as cartilage replacing implants as well as replace other soft body parts. It can be seen that the addition of ZnO NPs up to 1 wt.% raises *E* for the UV-cured samples, where *E* remains essentially constant for higher concentrations of the NPs. The incorporation of ZnO NPs had no effect on the value of *E* for the DLP-printed samples, making this method more predictable and reliable. 

A clear rise in the ultimate tensile strength and maximum elongation can be seen in the DLP prints for all ZnO NP concentrations; however, this tendency is not always so clear and definite. We also identified that the concentrations over which the incorporation of additional ZnO NPs causes more mechanical defects to the product, rather than strengthening it, are different according to the polymerization technique used. This is why the best elongation and ultimate tensile strength for DLP were found at 2 wt.%, but only at 1.5 wt.% for the UV-cured samples. Nevertheless, 1.5 and 2 wt.% of ZnO NPs resulted in a strong and elastic polymer, which can withstand relatively high stresses and strains. 

The differences in mechanical properties between the two vat polymerization processes can be attributed to the concentration of TPO (the photoinitiator) in the solutions, the strong absorbance of ZnO, the light beams’ focus, and the light intensities applied. Generally, a reduced amount of photoinitiator can result in improved mechanical properties as fewer and longer chains form during polymerization. For DLP printing, less TPO had to be added, explaining why *E* did not change between resins. The strong light absorbance of ZnO and the light intensities used can also contribute to the differences between the DLP printed and UV-cured samples, as the light intensity decreases through the mold (UV-curing) as it is absorbed by the NPs. When using DLP, however, the layer-by-layer movement of the bed and the beam focus by the voxel assures a similar light intensity throughout the polymerization. This is why we expect to observe certain limitations regarding the strength of large UV-cured implants when upscaling the process for bigger parts that are unlikely to occur for printing.

Therefore, thanks to their superior ultimate tensile strength and their repetitive *E*, as seen in Table 2, as well as the numerous ways to alter such properties, we conclude that DLP-printed implants are more promising than UV-cured samples and that the optimal concentration of ZnO NPs in the samples should vary between 1.5 and 2.0 wt.% to obtain the best mechanical properties. 

### 3.4. Antibacterial Activity

**Figure 3:**
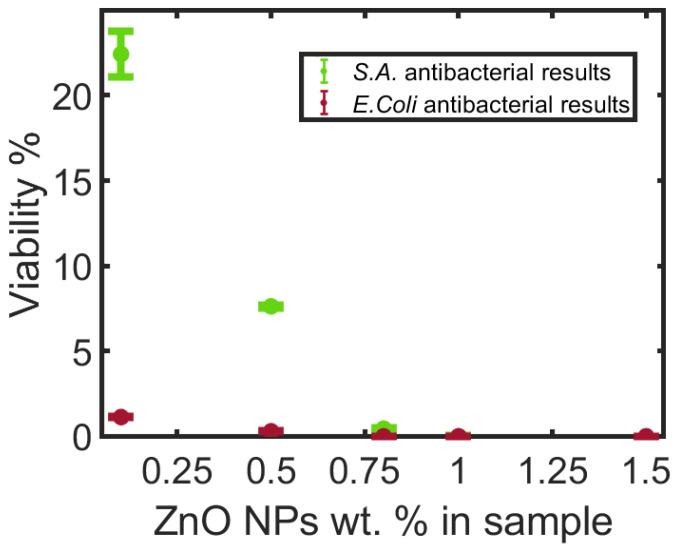
*S.A.* and *E. coli* viability percentage for different ZnO NP wt.% in UV-cured samples.

**Figure 4:**
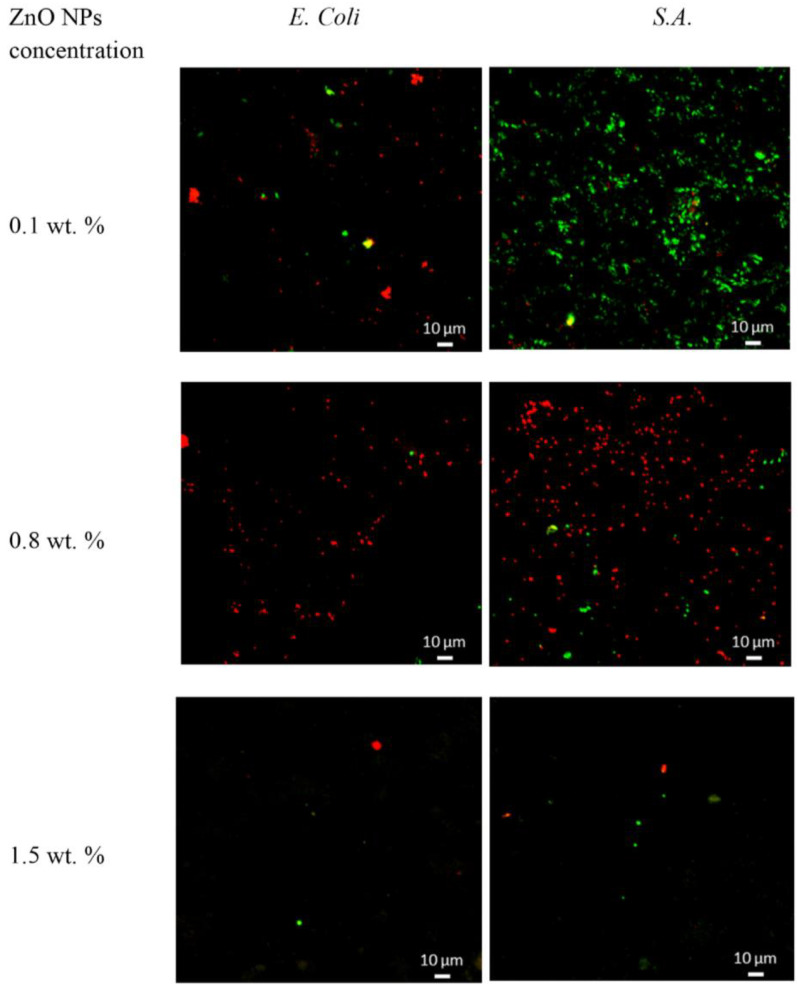
LIVE/DEAD microscopy scan of samples with different ZnO NP concentrations. Live and dead bacteria are colored green and red, respectively.

The minimum inhibitory concentration (MIC) of ZnO NPs was investigated using viability percentage for both UV-cured and 3D-printed samples by applying a turbidity method, using PEGDA samples containing no ZnO as control groups for bacteria growth under incubation on the hydrogel surface. Yet, since the fabrication of UV-cured samples was faster and the antibacterial tests required a relatively large number of samples, we report the results obtained for the UV-cured samples here (Figure 3). It is worth mentioning that similar results, although with lower accuracy, were obtained with the 3D-printed samples. Further confirmation of the antibacterial activity was achieved using the LIVE/DEAD method for different concentrations of ZnO NPs (Figure 4).

Figure 3 shows that the antibacterial activity of the UV-cured samples improves with the increasing concentration of the ZnO NPs. The MIC cannot be determined as a single ZnO NP concentration but ranges between 0.8 and 1 wt.% of ZnO NPs for *S.A*. and is somewhat lower for *E. coli*. By working above this range, we observed high antibacterial activity in samples, regardless of the vat polymerization method used. 

The LIVE/DEAD measurements (Figure 4) show some very interesting features. Not only does the eradication of the bacteria improve by increasing the concentration of the ZnO NPs in the samples, but in general, fewer bacteria (living or dead) can be detected in the samples. At high concentrations of ZnO NPs, hardly any bacteria, living or dead, are observed, as expected from direct contact with ZnO NPs. We did not anticipate obtaining such extremely high antibacterial activity on these surfaces, which is very encouraging for further applications.

The same procedures were performed for both UV-cured and DLP-printed samples (1.5 wt.% ZnO NPs) with different ratios of PEGDA and various additive monomers, e.g., methyl methacrylate, for both *S.A*. and *E. coli*. As before, strong antibacterial activity was observed for all measured samples, implying that the antibacterial activity is independent of the other constituents of the samples besides the ZnO NPs. It is worth mentioning that the almost complete absence of dead bacteria on the ZnO-NP-embedded surfaces is very encouraging because it has been reported that by providing available nutrients, dead biomass can be a factor that may affect pathogen regrowth [44]. In other words, the observed eradication of the bacteria negates the possible formation of biofilms on top of dead bacteria film and, thus, the overall reduced antibacterial activity of the surface over time.

## 4. Conclusions

We compared the mechanical properties and antibacterial activities of PEGDA-based samples prepared by UV curing and 3D printing (by DLP) in which ZnO nanoparticles (NPs) were incorporated. Both vat polymerization samples were tested for their antibacterial activity by exposure to *S.A*. and *E. coli* bacteria. We found that 1.5–2.0 wt.% of ZnO NPs is optimal for processing good resins for both UV-cured and DLP-printed medical applications such as implants. ZnO NPs seem to offer several advantages, such as a long-lasting antibacterial effect, which is presumably due to the strong entrapment in the polymer matrix. While both vat polymerization techniques proved to provide strong and elastic results, DLP printing showed greater elasticity and produced sturdier products. Future efforts for the usage of PEGDA-based resin for medical applications should attempt to improve even further the performance of the 3D-printed implants by modifying the PEGDA and incorporating additional functionalities via the use of various precursors. Better control and understanding regarding the composition of the structure should be the first research goal in upscaling the production of PEGDA-based materials for widespread medical applications. Possibly, mixing the resin while printing or regularly adding more freshly made resin could further improve the process moving forward. A more consistent concentration of ZnO NPs could be kept this way should the competitive aggregation of the NPs prove to be a problem for longer prints.

## Figures and Tables

**Table 1:** EDS analysis of the atomic and weight percentages of the three most detected elements (excluding Ir) in both 3D-printed and UV-cured samples containing 1.5 wt.% ZnO NPs.

Sample	Atom	Atomic %	Weight %
DLP-printed	Zn	0.4 ± 0.1	2.0 ± 0.2
	C	58.1 ± 0.3	50.2 ± 0.3
	O	41.5 ± 0.4	47.8 ± 0.5
UV-cured	Zn	0.6 ± 0.1	2.8 ± 0.2
	C	62.5 ± 0.3	54.4 ± 0.2
	O	36.9 ± 0.3	42.8 ± 0.3

**Table 2:** Summary of Young’s modulus, maximum elongation, and ultimate tensile strength of both UV-cured and DLP-printed samples, as measured by tensile tests.

	Sample	No ZnO	ZnO NPs 1 wt.%	ZnO NPs 1.5 wt.%	ZnO NPs 2 wt.%
Young’s modulus, *E* (MPa)	UV-Cured	10.7 ± 0.1	11.62 ± 0.07	11.8 ± 0.5	11.9 ± 0.4
	DLP-Printed	11.7 ± 0.4	11.7 ± 0.6	11.7 ± 0.6	11.8 ± 0.3
Max. elongation (%)	UV-Cured	10.7 ± 0.7	10.2 ± 0.8	10.7 ± 0.6	8.3 ± 0.2
	DLP-Printed	9.85 ± 0.09	12 ± 1	10.9 ± 0.6	14.6 ± 0.6
Ultimate Tensile Strength (MPa)	UV-Cured	1.0 ± 0.1	0.87 ± 0.09	1.0 ± 0.1	0.88 ± 0.06
	DLP-Printed	0.90 ± 0.02	1.4 ± 0.1	1.12 ± 0.01	1.3 ± 0.3

## Data Availability

Relevant data is contained within the article or Supplementary Material.

## Supplementary Materials

The following supporting information can be downloaded at https://www.mdpi.com/article/10.3390/polym15173586/s1, Figure S1: Shape and dimensions of the doggybone sample for mechanical tests; Figure S2: Optical microscope images of the top, bottom, and cross-section planes of samples containing 1.5 wt.% ZnO NPs made by DLP printing (A,C,E) and UV curing (B,D,F). (A,B) Top view, (C,D) bottom view, and (E,F) cross-section view; Figure S3: Zn EDS mapping of the three most common elements in the samples made using DLP printing (A,C,E,G) and UV curing (B,D,F,H), both with a concentration of 1.5 wt.% ZnO NPs; Table S1: The final solution concentrations of the four most repeated concentrations of ZnO NPs; Table S2: The range of printing parameters applied. Specific parameters within these ranges were found for every instance of resin tested.
