# Supplementary material for: Vat Polymerization by Three-Dimensional Printing and Curing of Antibacterial Zinc Oxide Nanoparticles Embedded in Poly(ethylene glycol) Diacrylate for Biomedical Applications
Source: Polymers (Basel). 2023 Aug 29;15(17):3586. doi: 10.3390/polym15173586 (PMC10490083; doi:10.3390/polym15173586)
Supplement: Supplementary file 1 [file polymers-15-03586-s001.zip › polymers-2583570-supplementary.pdf]

Supporting Information

Vat Polymerization by 3D Printing and Curing of Antibacterial Zinc Oxide Nanoparticles Embedded in Poly(Ethylene Glycol) Diacrylate for Biomedical Applications

Guy Naim, Netta Bruchiel-Spanier, Shelly Betsis, Noam Eliaz and Daniel Mandler*

[1]Institute of Chemistry, The Hebrew University of Jerusalem, Jerusalem, Israel 9190401

[2]Department of Materials Science and Engineering, Tel-Aviv University, Ramat Aviv, Israel 6997801

Supplementary Figures

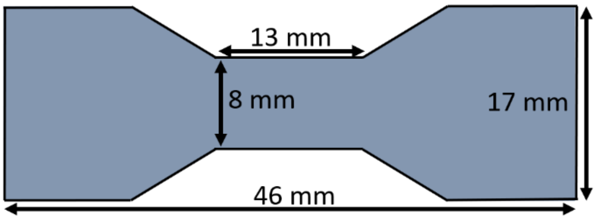

Figure S1. Shape and dimensions of the doggybone sample for mechanical tests.

Figure S1 shows the shape and dimensions of the doggybone shape samples used for mechanical testing. The same shape was used both as the model for 3D printing and as a mold for UV-curing.

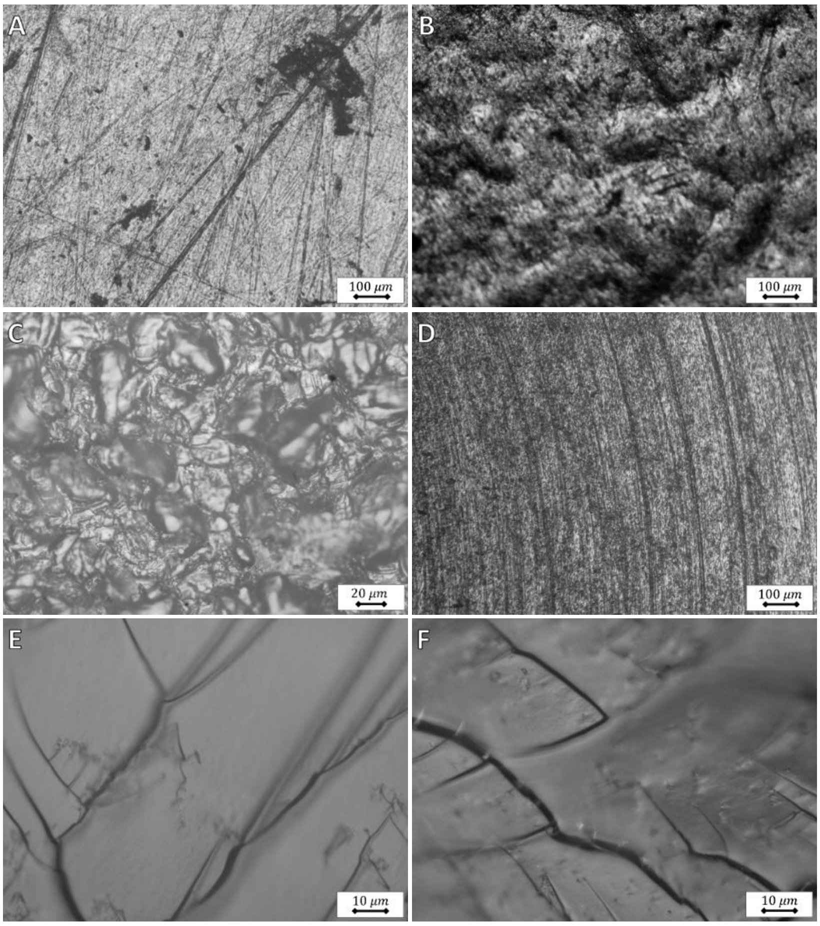

Figure S2. Optical microscope images of the top, bottom, and cross-section planes of samples containing 1.5 wt. % ZnO NPs made by DLP printing (A, C, E) and UV curing (B, D, F). (A, B) Top view, (C, D) bottom view and (E, F) cross-section view.

Figure S2 shows the 3D printed and UV-cured samples' top, bottom, and cross-section views. The top surface of the samples (Fig. S2 A, B) was in contact with the metallic substrate during 3D printing or was exposed to air during UV-curing. These surfaces match well the vat polymerization method used; namely, the surface of the DLP-printed sample is scratched from past uses, resulting in obviously identical marks on all of the DLP-printed samples. The UV-cured samples have the flattest surface obtained due to the flattening effect of the fluid precursor solution. The bottom side of the printed sample (Fig. S2 C) is flat but uneven, supposedly from small imperfections of the transparent bottom where the process took place. The bottom of the UV-cured sample (Fig. S2 D) shows circular lines as a result of the mold topography. The two cross-section images (Fig. S2 E, F) look similar, with large grains and grain boundaries.

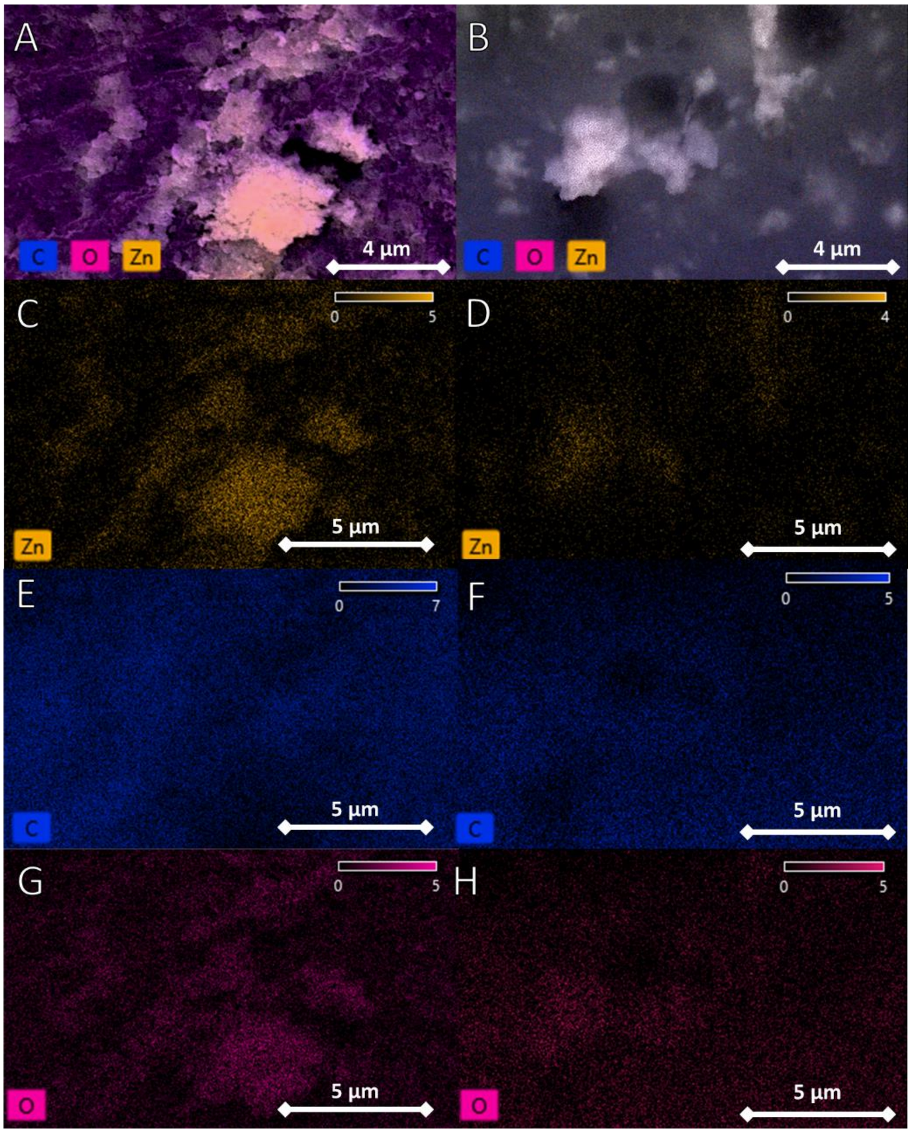

Figure S3. Zn EDS mapping of the three most common elements in the samples made using DLP printing (A, C, E, G) and UV curing (B, D, F, H), both with a concentration of 1.5 wt. % ZnO NPs.

Table S1. The final solution concentrations of the four most repeated concentrations of ZnO NPs.

	UV curing (wt. %)				DLP printing (wt. %)			
PEGDA	80	80	80	80	80	80	80	80
TDW	19.9	18.9	18.4	17.9	19.985	18.985	18.485	17.985
ZnO NPs	0	1	1.5	2	0	1	1.5	2
TPO	0.1	0.1	0.1	0.1	0.015	0.015	0.015	0.015

Table S2. The range of printing parameters applied. Specific parameters within these ranges were found for every instance of resin tested.

Wavelength	405 nm
Slice Thickness	0.05 ± 0.02 mm
Light Intensity	28.8 mW/cm^2
Exposure Time	1.3 ± 0.5 s
Fill Exposure	100 %
Separation Velocity	4.00 ± 1.0 mm/s
Separation Distance	4.9 ± 0.3 mm
Approach Velocity	4.00 mm/s
